# Extremists of a feather flock together? Community structures, transitivity, and patterns of homophily in the US Islamist co-offending network

**DOI:** 10.1371/journal.pone.0298273

**Published:** 2024-06-05

**Authors:** Anina Schwarzenbach, Michael Jensen

**Affiliations:** 1 Belfer Center for Science and International Affairs, Harvard University, Cambridge, MA, United States of America; 2 Department of Criminology and Criminal Justice, University of Maryland, College Park, Maryland, United States of America; 3 National Consortium for the Study of Terrorism and Responses to Terrorism, University of Maryland, College Park, Maryland, United States of America; The Ohio State University, UNITED STATES

## Abstract

Prior research suggests that members of terrorist groups prioritize forming network ties based on trust to improve their organizational and operational security. The homophily principle, which postulates that individuals tend to form relationships based on shared characteristics, can be a key mechanism through which people identify trustworthy associates. Next to homophily, the mechanism of establishing interconnected relationships through transitivity is also well-known to serve this purpose and shape community structures in social networks. We analyze the community structures of the Islamist co-offending network in the United States, which is highly violent, to assess whether homophily and transitivity determine which extremists form co-offending ties. We rely on a new database on the individual attributes and the co-offending relationships of 494 Islamist offenders radicalized in the United States between 1993 and 2020. Using community detection algorithms, we show that the US Islamist co-offending network is highly clustered, modular, and includes many small but only a few large communities. Furthermore, results from exponential random graph modeling show that transitive relationships as well as spatial proximity, ideological affiliation, and shared socio-cultural characteristics drive co-offending among US Islamist extremists. Overall, these findings demonstrate that the processes of homophily and transitivity shape violent social networks.

## Introduction

Research [1–5] has shown that the social structures of extremist networks influence how decisions are transmitted between actors and they can significantly impact how network members behave. Ties between actors at the local level not only define the structures of terrorist cells but also influence the global structure of extremist ecosystems [6, 7]. For example, by yielding clusters of different densities, local networks define whether extremist movements are highly or loosely organized, thus impacting the rate at which propaganda and tactical decisions are transmitted across groups and movements [8]. Moreover, studies [9, 10] have found that small local networks that display high degrees of in-group cohesion are susceptible to group biases, echo chamber effects, and other processes that make it more likely that network members will engage in acts of violence.

Social network analysis (SNA) has become an increasingly important research method for studying extremist networks generally and local extremist networks specifically. Much of this research has focused on exploring how networks influence outcomes, such as rebel alliances [11], conflict between militant groups [12], and insurgency and counterinsurgency [13, 14]. Research relying on SNA has paid far less attention to analyzing how relationships are formed in extremist networks. Why do some extremists form ties with each other while others do not? Which local network structures and individual covariates increase the probability of forming a tie? And finally, which community structures—clusters of individuals within the network that are densely connected to each other, and less to other individuals in the network—do these ties produce? This study addresses these questions, paying particular attention to the roles of homophily—the tendency for people to form ties to others who are similar to themselves—and transitivity—the tendency for two people to form a connection if they share a mutual contact—as key mechanisms in the formation of co-offending relationships and communities in the US Islamist extremist network. We analyze how co-offending relationships in the Islamist extremist network are formed as a function of both the local network structure and individual covariates, and ask whether and how homophily and transitivity shape co-offending relationships and communities.

Our analysis follows on research about the security-efficiency trade-off in illicit networks [15, 16], which finds that extremist groups’ pursuit of ideological motives differentiates them from other types of illegal organizations and makes it more likely that they will prioritize forming relationships that increase group security over group efficiency. While groups that are driven mainly by material incentives often sacrifice security to make their networks more efficient and profitable [17], the focus on security in extremist groups puts constraints on who is allowed to join the network and how many ties are ultimately formed [18]. While extant research has demonstrated the prioritization of security in terrorist groups, it has not identified the mechanisms through which extremists identify and form trusted partnerships. We contribute to the literature on terrorist recruitment and group formation by exploring how terrorists form co-offending relationships that increase the group’s organizational and operational security. We argue that the principle of homophily—the idea that “similarity breeds connection”—acts as a primary mechanism that determines the ties that are formed in extremist groups [19]. Homophily increases the internal security of illicit groups by ensuring that the ties that are formed are based on shared experiences, common norms and values, and mutual trust. In addition to homophily, transitivity is an important driver of co-offending relationships in Islamist extremist networks. While homophily emphasizes similarity as the driving force behind building secure connections, transitivity highlights the interconnectivity and informal vetting within networks that increase members’ trust in each other. Both shape community structures within social networks and contribute to the emergence of cohesive groups within a network.

In this paper, we focus on the US Islamist extremist network, which is a part of the broader Salafi-Jihadist movement. The jihadi militant ideology is practiced by Islamist Salafists who seek the immediate overthrow of incumbent regimes and the non-Muslim geopolitical forces which support them, paving the way for an Islamist society that would be developed through martial power. Among extremist networks, those formed by Salafi Jihadists are especially violent and produce comparatively high causality rates [20–22]. Jihadi extremism has a history of inspiring terrorist attacks at the local level. According to the Global Terrorism Database (GTD) [23], from 2013 to 2019 there were a total of 29 ISIS-inspired attacks in the United States. These attacks include the San Bernardino shooting in 2015 and the 2016 Orlando nightclub attack, which remains the deadliest terrorist attack on US soil since 9/11.

In this study, we use a definition of co-offending that includes all instances where two Islamist extremists (1) co-conspired in the same plot or crime and/or (2) communicated with each other for the purposes of organizing; training; promoting extremist content; or transferring knowledge, money, or materials before they committed separate crimes motivated by an extremist Islamist ideology. Thus, our definition of “co-offenders” is purposefully broad and includes individuals who perpetrated crimes together and those who communicated with each other but committed separate crimes. We use this conceptualization to capture the fact that lone actors are very rarely, if ever, lonely actors [24, 25]. Rather, those who perpetrate terrorist crimes on their own are often embedded in networks with like-minded offenders who share the operational and tactical knowledge that make successful acts of terrorism possible. Thus, in order to fully understand how violent networks form and achieve their goals, it is important to consider relationships beyond those that are simply made up of people engaged in the same terrorist plots.

Studies that have applied SNA to explore Islamist co-offending networks have focused mainly on exploring the density and centrality measures of the network and describing key actors and their roles. Krebs [26] famously explored the 9/11 hijacking network, relying on network information extracted from newspaper reports. The results revealed a dense and highly connected core network, where key individuals involved in the plot were connected through kinship, ties forged in school and shared experiences. Reynolds and Hafez [2] analyzed the foreign fighter phenomenon in Syria and Iraq, finding that peer-to-peer networks were the most important mobilization factor for German foreign fighters [see also 27–29, for similar conclusions]. And Nesser et al. [30] demonstrated that local networks played key roles in facilitating terrorist attacks in European countries. The few studies that have explored the formation of relationships among Islamist offenders have not included information on the individual attributes of the network members [15, 31, 32], possibly because of the difficulties of gathering comprehensive data on Islamist extremist actors. Those that have included individual-level information [33–35] have not explored the role of homophily in predicting the probability of co-offending ties in Islamist extremist networks.

Identifying how Islamist co-offending relationships are made and how the network is clustered is an important part of preventing future acts of violence. Research on radicalization suggests that it is often hard to identify the relatively small subsets of potentially violent individuals from the much larger populations of ideologues and propagandists [36–39]. Thus, disrupting Islamist terrorist plots and stemming the tide of terrorist recruitment requires sifting through considerable noise to identify networks of mobilized offenders and find weak points through which the networks can be dismantled. The rise of online communication technologies and the spread of extremist propaganda in digital spaces has created unprecedented opportunities for terrorists to meet, form relationships, and potentially co-offend. Much of the global effort to prevent acts of terrorism now focuses on disrupting the formation of digital relationships through content moderation, account takedowns, and law enforcement surveillance of online communities. However, while digital communities are good at fostering the formation of shared extremist beliefs, they are inherently insecure. Trusted narrators can be hard to identify and vetting mechanisms for entry into virtual communities are often weak or nonexistent. Terrorists are aware of these limitations and must consider the potential negative consequences of forming online relationships when plotting attacks. We argue that when it comes to networks of co-offenders, extremists prioritize forming relationships based on trust over those that are more readily available but less secure. For practical and strategic reasons, including the need to avoid detection, terrorists prefer trusted co-offending relationships that are based on local interactions, shared socio-cultural experiences, and common ideological goals.

For the analyses presented in this paper, we rely on a new database—the Social Networks of American Radicals (SoNAR) [40]—which includes information on the dyadic relationships between US Islamist offenders. SoNAR also includes data on the attributes of network members, including socio-economic background measures, location of radicalization, year of radicalization, terrorist group affiliation, and the specific roles the offenders played in their respective networks (e.g., facilitator, recruiter, mentor, etc.). Although there are several Islamist-Salafist thinkers (e.g., Muhammad Nasiruddin al-Albani) who do not advocate for violent military strategies to achieve their goals, in the US context, individuals classified as jihadists in SoNAR are typically connected to, or inspired by, violent Islamist-Salafist groups. In the context of this study, the term Islamist extremist is used to refer to individuals who subscribed to a jihadist militant ideology.

Using community detection algorithms and exponential random graph models (ERGMs), we analyze the US Islamist co-offending network. The results reveal a highly clustered and modular network, featuring many small communities with high within-community cohesion. Moreover, we detect robust patterns of transitivity and homophily in the US Islamist co-offending network. Controlling for the year an individual engaged in illegal activities linked to Islamist extremist ideology and other relevant predictors, we find that sharing the same country of origin and spatial proximity greatly increase the likelihood that two Islamist extremists will co-offend. Ideological affiliation also helps in understanding co-offending relationships: members of the same terrorist group are more likely to form co-offending ties. Furthermore, we find that religious converts and non-converts are also more likely to co-offend within their respective groups and that kin- and friendship influence the formation of co-offending relationships.

The findings from this study shed new light on community structures in Islamist extremist networks and help explain which factors facilitate co-offending relationships. Our findings on the prevalence of transitivity and patterns of homophily rooted in local structures and based on shared socio-cultural characteristics point to the importance of trusted relationships when individuals decide to commit crimes together. They also suggest that individuals prefer to rely on trusted personal and local relationships instead of relationships built in the digital environment when searching for potential collaborators. Therefore, counterterrorism efforts in digital spaces should complement rather than replace traditional policing at the local level. Ultimately, understanding how co-offending ties are formed may not only help prevent acts of terrorism at the local level, but given the interconnection between local and global networks, it may also help combat the global spread of extremist movements.

### Homophily, transitivity, and community structures

Research [41, 42] has found that similarity increases the probability of co-offending in social networks. For example, studies [43, 44] have linked identity-related measures, such as country of origin, to levels of terrorist activity and co-offending, while more general studies of crime [45–47] have found that co-offending is greater among people of the same age, gender, and ethnicity. Individuals typically seek content and meaning when connecting [48, 49]. Shared characteristics provide this and they are interwoven with collective narratives, which have been found [50] to provide a strong rationale in extremist networks for an individual’s evolution toward violent action.

As a result of their focus on security, co-offending networks prioritize similarity as a means of identifying trustworthy associates and potential recruits. Given intense law enforcement attention, terrorists are likely to form close-knit groups where members act as gatekeepers, admitting only those whom they think they can trust [9, 10]. Three shared characteristics are particularly important to the processes of forming extremist co-offending ties: shared socio-cultural backgrounds or experiences; geographic proximity; and common affinity for the same terrorist group or ideology. We focus on these similarities as opposed to others, like similar occupations or educational levels, because they are directly related to the formation of secure relationships that are built on trust. First, extremists are more likely to form co-offending relationships with those with whom they have shared socio-cultural backgrounds or experiences. Holman [51] and Magouirk et al. [33] found that when recruiting new members, extremist organizations are more likely to focus on individuals with whom they have kinship ties or those who are a part of common religious or diaspora communities. Similarly, Piazza [52] found that domestic terrorist activity in response to socioeconomic discrimination is often committed by members of the same ethnic communities. Basing co-offending ties on shared socio-cultural experiences and backgrounds allows extremist groups to separate trustworthy recruits from those who seek to infiltrate and undermine the organization, thus increasing the security of their networks. Second, extremists are more likely to form co-offending ties if they live in physical proximity to each other. Indeed, research focusing on co-offending networks [41, 53] also found that spatial proximity is a main driver of co-offending relationships. Sageman’s [35] “bunch of guys” thesis notes that Islamist extremist networks in Western countries tend to be made up of individuals who live in the same communities, occupy the same social spaces, and pray at the same mosques. Similarly, analyses of foreign fighters from Germany [2], the Netherlands [54], Canada [55], Australia [55], and Sweden [28] found that the fighters tended to be mobilized within local networks. Being from the same physical location not only increases the chances that two individuals with shared extremist beliefs will encounter one another, and it also acts as a key vetting mechanism by allowing individuals to tap into their local social networks to gather information on a potential co-offender and assess their trustworthiness. By contrast, jihadists who rely on online communities to form relationships may not be connected to trusted associates who can accurately judge the character of potential recruits. This is especially concerning in the US context, where individuals looking to form relationships with fellow extremists online often unknowingly connect with undercover law enforcement [56]. Finally, extremists are more likely to form co-offending ties if they share affinities for groups or movements with similar values and goals. The formation of networks along ideological lines is to be expected considering that not all Jihadist groups have the same values and goals. Some are motivated by global concerns, while others are more interested in local matters. Some adhere to a strict interpretation of Islam, while others are more progressive in their views. Some are highly violent and pursue attacks outside of conflict zones, while others prioritize attacks on military targets or government personnel in areas where civil conflicts are present. Extremists, like most people, are more likely to seek relationships with others who share their interests and values. Research shows that terrorist plots frequently involve members of the same terrorist group [23]. Moreover, it is well documented that individuals in extremist groups often compete, rather than collaborate, with people in other terrorist groups, leading to patterns of inter-group conflict and increased violence—a process known as “outbidding” [57–59]. The studies [60–62] that have observed patterns of cooperation and co-offending among terrorist groups note that this type of collaboration typically only involves groups with similar ideologies, frames, and organizational structures [63].

US-Jihadists who do not have access to local extremist networks made up of members with whom they are familiar, or with whom they have shared socio-cultural backgrounds, experiences and common goals, are unlikely to form co-offending ties with other extremists. Instead, they are likely to pursue their terrorist ambitions on their own or look to online communities for support, where they are at a greater risk of detection by law enforcement. In the US context, many of the Islamist extremists who seek to form co-offending ties in online communities are converts to Islam who lack access to local networks based on kinship or diaspora ties. Indeed, although converts to Islam represent a small percentage of the Muslim community in Western countries, when it comes to Islamist extremism, they are considerably overrepresented [64]. Research [65, 66] has found that converts to Islam have participated in major Islamist terrorist plots and attacks. When successful in forming co-offending relationships, converts are likely to connect with other converts who are driven by the same ideological motivations, come from similar social and ethnic backgrounds, and have undergone similar radicalization processes [67, 68].

In addition to homophily, transitivity is a key mechanism in forming social networks based on trust and security [69, 70]. Transitivity suggests that any two Islamist extremists are more likely to co-offend if an indirect tie already exists between them. Mutually trusted third parties facilitate new co-offending relationships by introducing prospective associates to each other and vouching for their trustworthiness. Thus, high levels of transitivity contribute to the formation of close-knit groups in social networks [71], including extremist groups [69]. We expect that Islamist co-offending takes place in networks made of dense, small clusters of interconnected individuals who act as gatekeepers that facilitate relationships between those they trust and guard the network from those they do not.

The processes of homophily and transitivity lead to the formation of intense bonds within terrorist groups, improving their security but also making their members susceptible to cognitive biases that increase the chances they will participate in violent extremism. Borgatti and Foster [72], for example, found that interactions within close-knit groups over time produce dense networks with narrow and homogeneous beliefs, social norms, and attitudes that can lead to extreme or risky behaviors. In an analysis of the al-Qaeda network, Sageman [35] showed how close-knit local cells also commonly engaged in a process of one-upmanship, causing the network’s clusters to become increasingly insular and violent, all whilst expanding their loyalty to the group itself. Similarly, LaFree et al. [73] found that membership in a close-knit group was the strongest predictor of participation in violent extremism when analyzing a sample that included violent and nonviolent extremist offenders. Close-knit groups are often formed by close friends or other individuals with strong emotive ties. Indeed, social learning perspectives [74] emphasize how peers transmit delinquent behaviors to others through a process of socialization.

## Materials and methods

### Data

In this study, we were interested in exploring co-offending relationships and community building among extremists radicalized in the United States who have espoused the Islamist extremist ideology through 2020. To be included in our study, an individual had to radicalize within the United States; that is, their radicalization process had to significantly advance while they were located within the territorial boundaries of the United States. “Significantly advanced” was determined on a case-by-case basis but generally meant that most of the individual’s radicalization occurred while residing in the United States. In addition to having radicalized in the United States, the individual had to espouse Islamist extremist ideological motives and committed at least one criminal offense linked to their promotion of Islamist extremist beliefs. Such evidence included being arrested, indicted, convicted, or killed because of an illegal act linked to the ideology, as well as joining an Islamist terrorist organization abroad. We included individuals who plotted or committed violent attacks, as well as those who committed non-violent criminal offenses, such as sending money or equipment to foreign terrorist organizations. We considered all individuals for whom Islamist extremist ideological motives were the prime driver of their engagement in illegal behavior—independent of whether they were part of a group.

We assigned a tie between two individuals if they were co-offenders in the same crime or if they exchanged information, tactical knowledge, or expertise before perpetrating separate crimes. Individuals in the data could be linked to more than one criminal event. A small number of actors had ties to different groups of Islamist offenders over time. For all Islamist extremists included in the study, co-offending involved a symmetric relationship (undirected network). The act of committing a criminal offense together implied that the Islamist extremists were engaged in a two-way communication or interaction. Lee and Butts [75] have found that symmetric relationships best reflect organizational membership and provided a more accurate measurement of network structure. Moreover, a co-offending relationship is typically the result of stable social ties. Goodreau et al. [76] have concluded that stable social ties are typically based on two-way communication. Each relationship was coded only once: if an individual had co-offending relationships with more than one individual in the data, then those relationships were coded in separate rows. Most of the Islamist extremists included in the network were affiliated with a (formal or non-formal) organization. Individuals who were inspired by a terrorist group without ever communicating with actual group members abroad were also coded as being affiliated with the group since their criminal offenses were motivated by their identification with the organization.

The data used in this study come from the Social Networks of American Radicals (SoNAR) database [77], a database on co-offending relationships between US extremists, which is a part of the Profiles of Individual Radicalization in the United States (PIRUS) project. PIRUS is a suite of cross-ideological, open-source datasets that include information on political extremists who radicalized in the United States, including offenders who committed crimes that were motivated by Salafi Jihadism. The datasets that make up the PIRUS project aim to capture the relevant aspects of individuals’ backgrounds and experiences that could potentially explain their radicalization trajectories. SoNAR contributes to the PIRUS project by recording co-offending relationships between US extremists. We matched the SoNAR data to the original individual-level PIRUS data to retrieve information on both co-offending relationships among Islamist extremists, as well as individual attributes of the Islamist offenders. The data come primarily from court records. In the United States, most of the Islamist extremist cases have been prosecuted at the federal level, where court documents are publicly accessible. Court documents included detailed information on the individual attributes of offenders and reported key relationships to other individuals relevant to the cases. Co-offending relationships were reported in court documents, as they were a key element in prosecuting the cases. To assess robustness, these data were compared with information on the cases retrieved from news articles and other publicly accessible reports. Islamist extremist cases were highly publicized in the media, and individuals directly involved in the cases were mentioned in news reporting. Thus, we are confident that we captured most– if not all—of the relationships in the US Islamist co-offending network.

We followed Diviák’s [1] approach to set the boundaries in criminal networks and focused on local, rather than global, ties between actors in the extremist networks. Consequently, we focused on the relationships between US-based Islamist offenders and did not include ties between individuals radicalized in the United States and individuals radicalized abroad. We made this decision for three reasons. First, the focus of this paper is the analysis of communities, transitivity, and homophily in the US Islamist co-offending network. Research [7, 8] found that local interactions and networks are key to understanding global networks. They shape the structure, dynamics, and relationships in such networks. By focusing on the local US context, we were able to collect comprehensive and trustworthy information that allowed us to analyze the impact of individual attributes on the formation of the Islamist co-offending network. Second, Krebs [26] has shown that while the inclusion of a wider set of international actors can make the resulting networks denser, transitivity—a key measure in our analyses—remains stable when compared to a network that only includes local actors. Third, the online and secretive nature of foreign communications makes collecting data on international ties very challenging and, in some cases, impossible. By focusing on the United States, we were able to achieve a comprehensive network that included all publicly known co-offending relationships among Islamist extremists.

We included all publicly identified Islamist extremists who committed crimes in the United States from 1993 to 2020. The decision to include cases across multiple decades was made to ensure that we had enough data to facilitate a social network analysis and to capture the formation of relationships across groups and time periods. For instance, several offenders in the data who were linked to al-Shabaab from 2006–2009 later formed ties to individuals with links to ISIS from 2014 to 2016—a dynamic that would be missed if we only used data from the most recent decade. However, we assumed that two individuals were more likely to form a co-offending tie if they were operating in close temporal proximity to each other, and thus we included a control variable for year in our models to account for the effect of time on the formation of co-offending relationships.

The summary statistics of the data on US Islamist co-offenders used in this study are reported in Table 1. The initial US Islamist extremist network had 711 nodes and 963 edges. We removed 217 nodes from the network. These were individuals with no connections in the US Islamist extremist co-offending network. They carried out the crimes by themselves and were not involved in any other form of co-offending relationship with anyone else in the network (such as a facilitator of an Islamist offense). We also excluded individuals who radicalized in the United States but only had co-offending ties with other individuals outside the US Islamist extremist network. The final US Islamist co-offending network used in this study had 494 nodes and 963 edges.

**Table 1:** Summary statistics.

Variable	Count	Min	Max	Per
Gender	494	1	2	
… men	461			93.3%
… women	33			6.7%
Terrorist group affiliation	494	1	9	
… al-Qaeda	152			30.8%
… ISIS	134			27.1%
… al-Shabaab	57			11.5%
… Hezbollah	49			9.9%
… Taliban	25			5.1%
… no formal organization	23			4.6%
… Hamas	17			3.4%
… other	32			6.5%
… unknown	5			1.1%
Year of exposure	494	1	6	
… 2001 to 2005	113			22.9%
… 2006 to 2010	133			26.9%
… 2011 to 2015	131			26.5%
… 2016 to 2020	57			11.5%
… before 2001	9			1.8%
… unknown	51			10.4%
Country of origin	494	1	11	
… United States	136			27.5%
… Somalia	63			12.7%
… Pakistan	33			6.7%
… West Bank and Gaza Strip	26			5.3%
… Lebanon	19			3.8%
… MENAT	63			12.7%
… Asia	31			6.3%
… Europe	22			4.5%
… America	15			3.0%
… Sub-Saharan Africa	10			2.0%
… unknown	76			15.5%
Location of exposure	494	1	15	
… New York	83			16.8%
… Minnesota	62			12.6%
… Texas	43			8.7%
… Florida	37			7.5%
… Michigan	33			6.7%
… California	32			6.5%
… Virginia	27			5.6%
… New Jersey	24			4.9%
… Ohio	18			3.6%
… North Carolina	14			2.8%
… other Midwest	35			7.1%
… other Northeast	22			4.5%
… other South	17			3.4%
… other West	38			7.7%
… unknown	9			1.6%
Religious convert	494	0	1	
… no or unknown	379			76.7%
… yes	121			23.3%
Family members	494	0	1	
… no or unknown	356			72.1%
… yes	138			27.9%
Friends	494	0	1	
… no or unknown	397			80.4%
… yes	97			19.6%
US plot involvement	494	0	2	
… no	235			47.6%
… yes	118			23.8%
… unknown	141			28.6%

### Measures

#### Ties

The dependent variable *co-offending tie* was the probability that a tie was formed between US Islamist offenders. The network was undirected. As a result, there were no asymmetric ties in the data.

#### Individual attributes

*Gender* measured whether the individual is male or female. The Islamist extremist network included 33 females and 461 males.

**Fig 1:**
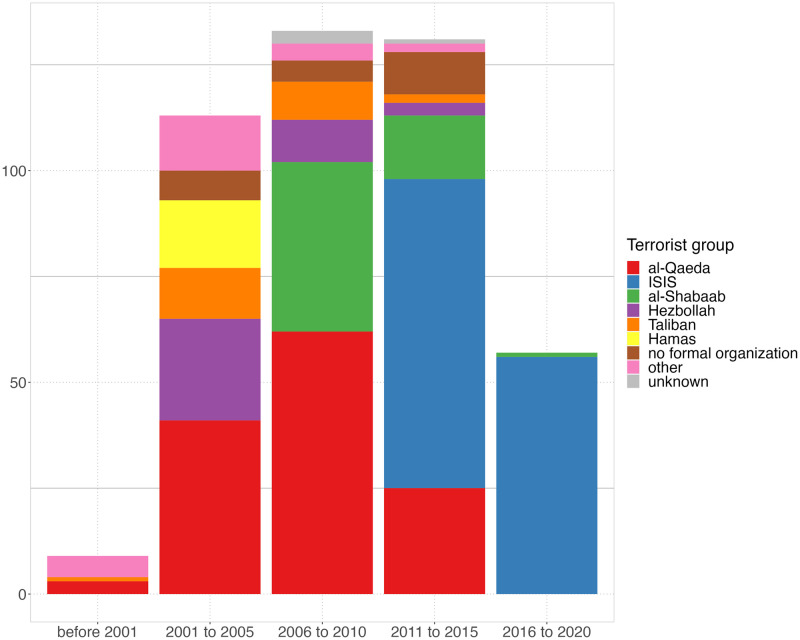
Counts of individuals radicalized within the same time period. Note: individuals whose year of exposure was unknown are excluded.

*Terrorist group affiliation* recorded the name of the existing designated Islamist terrorist organization or Islamist extremist group with which the individual was affiliated (see also Fig 1). It included organizations with which the individual self-identified, as well as those linked to the individual by authorities or credible media sources. We identified six different terrorist groups. The largest groups recorded in our data were the Islamic State of Iraq and Syria (ISIS) and al-Qaeda. Other prominent groups were al-Shabaab, Hezbollah, the Taliban, and Hamas. Al-Qaeda included individuals affiliated with al-Qaeda in the Arabian Peninsula (AQAP) as well as other al-Qaeda denominations. Individuals who were associated with organizations not featured above were assigned the category “other groups”. Individuals who were not affiliated with any formal organization were assigned a separate category “no formal organization”. For around one percent of the individuals, the affiliation was unknown.

*Year of exposure* designated the year in which the individual’s activity/plot first came to public attention. This usually matched the time of incident or arrest, or earliest mention of the individual in sources, so long as these were related to the plot/radicalization of the individual. We collapsed the years into 5-year buckets, see Fig 1, to account for the fact that individuals who radicalized within a few years of each other had a better chance of being connected.

*Country of origin* recorded the country of origin of first- and second-generation immigrants to the United States. A large share of individuals in the data originated from Pakistan, Somalia, Lebanon and the West Bank and the Gaza Strip. Other individual countries were aggregated into world regions. Where the country of origin was not known, the individual was assigned the category “unknown country of origin”. In addition to these values, the data include 136 Islamist extremists who were born in the United States.

*Location of exposure* was coded as the US state and city where individuals were living at the time that they committed their crimes. Islamist extremists have been reported in all US regions. Not surprisingly, they tended to feature in higher numbers in more populous and urban states. However, there were exceptions. For example, Minnesota had the second-highest number of US Islamist extremists, topped only by New York.

*Religious convert* measured whether the individual was a convert to Islam at the time of their criminal activity. Conversion might have occurred prior to, during, or after radicalization. About a third of the Islamist extremists in the data were converts.

*Family members*. This variable measured whether the individual had a familial relationship with at least one other individual in the network. It also included individuals who were in a romantic relationship with another individual in the network (husband/wife, boyfriend/girlfriend, etc.). In the US Islamist co-offending network, 138 individuals had family members in the network. Of those, 32 were the spouses or partners of another extremist in the network.

*Friends* measured if the individual had friends in the network with whom they had a positive, affective bond prior to engaging in Islamist extremism. Examples included attending high school with one another, family friends from childhood, or playing sports together before becoming involved in extremism. Such relationships indicated a closer bond that went beyond mere acquaintance. Out of the 494 extremists included in the US Islamist extremist network, 97 had friends in the network. The variables *Friends* and *Family members* were measured at the node level. Each individual in the data was assigned a yes or no value indicating whether they were friends prior to joining the network or had family members involved in the Islamist co-offending network.

*US plot involvement*. This variable informed whether the individual was involved in a plot carried out on US soil. Out of the 494 Islamist extremists, 118 planned or carried out violent actions within the United States.

### Analytic strategy

#### Community detection

We started with a description of the structural properties of the US Islamist co-offending network. We identified communities relying on the Walktrap community detection algorithm. These analyses were carried out with the statistical software R, mainly with the igraph package [78–80].

This algorithm measures the similarity between vertices based on random walks. It is well-suited to capture the community structure in a network and can efficiently identify dense subgraphs of sparse graphs [81]. Compared to other community detection algorithms, the Walktrap method has been found to fit real-world social network data particularly well [82], and is among the top-performing methods for uncovering the structure of small networks with less than 1000 nodes [83].

To measure the strength of a network’s subdivision into communities we relied upon the modularity measure from the Walktrap algorithm. Networks with high modularity have dense connections between nodes within communities, but sparse connections between nodes in different communities. As a sensitivity analysis, we also used Louvain as an alternative algorithm.

#### Exponential random graph models

Next, we computed exponential random graph models (ERGMs). The analyses were carried out with the statistical software R, mainly with the package ergm [80, 84–86]. ERGMs are a class of models concerned with explaining the patterns of ties in a social network. They allow the researcher to examine competing theories regarding the formation of network ties, all within a single analysis [87].

We chose to rely on ERGMs because we were interested in predicting co-offending ties among US Islamist extremists as a function of the local network structure and relevant individual covariates. Specifically, we were interested in testing two network theoretical concepts for tie formation in the US Islamist extremist network: transitivity and homophily. Moreover, given that we were utilizing observations from social networks with interconnected nodes, dependencies between the edges of the network were likely to arise. For instance, the likelihood of Islamist extremist offender A to co-offend with the Islamist extremist B may have been conditional on the fact that both A and B also co-offended with the Islamist extremist C. If there are dependencies between edges, failing to account for these dependencies may result in model misspecification and biased conclusions about the underlying network structure. ERGMs recognize the complex dependencies within relational data structures and allow for the inclusion of terms in the model that capture various network configurations and dependencies. They also account for the extent to which local selection forces shape the global structure of the network [84].

#### Model specifications

We performed ERGMs on an undirected cross-sectional network, excluding the isolated nodes. We were primarily interested in detecting transitivity and patterns of homophily in the network and specified a network with the following characteristics. The dependent variable was the *probability of forming a co-offending tie*. To investigate the effects of homophily in the network of US Islamist co-offenders, we included actor-relation effects (effects that include the attributes of the actors in the network). We also specified purely structural effects, following Robins and Lusher [87] recommendation that models should include at least a density parameter as well as some control over degree distribution and closure. These effects do not depend on the characteristics of the network nodes. Previous work [76] demonstrated the importance of including purely structural effects when analyzing homophily in social networks. Without them, measures of homophily risk being severely inflated.

The following purely structural effects were included in the ERGM specification: *edges*, geometrically weighted edgewise shared partner distribution (*gwesp*), and geometrically weighted degree distribution (*gwdegree*) [85]. *Edges* measured the number of edges in the network. We included this variable to control for the density of the network. *Gwesp* was the coefficient for transitivity and captured triadic closure. Its value indicate the likelihood that when two actors have ties to the same third actor, they will also create or maintain a tie. It places a lower weight on each additional shared partner. This geometric decay is governed by an *α* parameter which is set by the user. Values closer to 0 place more relative weight on edges with one shared partner and edges with any number of shared partners. We chose a geometric decay *α* = 0.25, following previous research using *gwesp* to model transitivity in social networks [88]. A positive *gewsp* coefficient means that there is a tendency for two actors who have ties to the same third actor to also create a tie. *Gwdegree* measured a node’s tendency to have multiple ties, placing a weight to the increasing number of connections each node has [85]. Again, the geometric decay of this variable is governed by an *α* parameter which is set by the user. Values close to 0 give more relative weight to smaller degree counts. We achieved model convergence by setting *α* = 0.25. A positive *gwdegree* indicates that there is a tendency of nodes in a network to preferentially connect to other nodes with higher degrees. This is a common structural property that is often included in ERGM specifications. As we were interested in assessing the role of transitivity on the propensity to form co-offending ties, all models included the transitivity coefficient *gwesp*. All models also included *gwedegree* as a control variable.

We also included the following actor-relation effects in the model specification: *nodematch*, and *nodefactor* [85]. Homophily or the propensity to form within-group ties was measured through the dyadic covariate *nodematch*. This variable measures homophily in a network based on a categorical variable. For the analyses presented in this paper, we used this measure to assess whether US Islamist extremists tended to co-offend when they shared a particular attribute. We measured the propensity to form homophilous (within-group) ties by including the parameter *nodematch* in separate models for the following attributes: *terrorist group affiliation*, *country of origin*, *location of exposure*, *religious convert*, *family members*, and *friends*. We also included two controls at the level of the individual attributes: *nodematch gender* and *nodematch year of exposure*. Previous research [89] pointed to gender preferences in forming co-offending relationships. Therefore, in the models we controlled for the *gender* of the Islamist extremist offender. Moreover, the Islamist extremists were likely to co-offend with those who were exposed to extremism around the same time. Thus, in the ERGM analyses, we controlled for this potential confounder by adding the *year of exposure*. For every *nodematch* included in the models, we also included the *nodefactor* with the same variable. Whereas *nodematch* is an interaction effect, *nodefactor* is the main effect of a categorical attribute. This proceeding allowed us to control for a potentially skewed distribution in the factor variables.

Results from the association matrix, see S1 Table in S2 File, suggested a medium to strong association between some of the variables included in the models. Therefore, we have taken a step-wise approach, exploring the effects of these variables in separate models. We present the results of the main ERG model specifications in Tables 2 and 3. We estimated six models.

1. M1 was the Bernoulli model that included only the structural term *edges*. This estimate can be interpreted as the baseline propensity for the occurrence of ties.
2. M2 was the baseline model and explored the within-group effects of ideological affiliation on the propensity to co-offend. To this aim, we included the homophily term for *terrorist group affiliation*. In addition, we included the purely structural effects *gwesp* and *gwdegree*. We also included the actor-relation controls *gender* and *year of exposure*.
3. In M3 we added the homophily term for *country of origin* to the baseline model M2 to investigate the effect of shared socio-cultural background on the propensity to form co-offending ties.
4. In M4 we added the homophily term *location of exposure* to the terms included in M2 as a proxy for the spatial proximity of the Islamist extremists included in the network. Again, we were interested in the effect of this variable on the propensity to form co-offending ties.
5. In M5 we estimated the effects of the homophily term *religious convert* as an alternative measure for ideological affiliation. We added this term to the baseline model M2.
6. In M6, we added the homophily terms *family members* and *friends* to M2 to test the effects of alternative measures of shared social characteristics.

**Table 2:** ERGM comparisons of the Bernoulli, terrorist group affiliation, country of origin and location of exposure models (M1 to M4).

	M1 Estimate (SE)	M2 Estimate (SE)	M3 Estimate (SE)	M4 Estimate (SE)
**Purely structural effects**				
*Transitivity*				
gwesp (fixed 0.25)		3.82(0.13)***	3.78(0.13)***	3.65(0.13)***
*Controls*				
edges	−4.83(0.03)***	−10.02(0.31)***	−9.60(0.42)***	−9.85(0.40)***
gwdeg (fixed.0.25)		2.61(0.20)***	2.91(0.21)***	2.99(0.22)***
**Actor-relation effects**				
*Homophily (key terms)*				
nodematch terrorist group affiliation		1.56(0.07)***	1.51(0.06)***	1.37(0.07)***
nodematch country of origin			0.65(0.06)***	
nodematch location of exposure				1.25(0.06)***
*Controls*				
nodematch gender		0.29(0.26)	0.28(0.27)	0.23(0.28)
nodematch year of exposure		0.85(0.06)***	0.79(0.05)***	0.78(0.06)***
nodefactor al-Shabaab		0.19(0.04)***	−0.20(0.07)**	−0.35(0.10)***
nodefactor Hamas		0.32(0.07)***	0.24(0.12)*	0.40(0.12)**
nodefactor Hezbollah		0.30(0.04)***	0.41(0.06)***	0.65(0.10)***
nodefactor ISIS		0.16(0.09)	0.07(0.11)	0.10(0.12)
nodefactor no formal org.		0.17(0.16)	0.15(0.16)	0.09(0.17)
nodefactor Taliban		0.41(0.06)***	0.43(0.06)***	0.33(0.10)**
nodefactor Asia			−0.01(0.15)	
nodefactor Europe			0.02(0.14)	
nodefactor Lebanon			−0.53(0.16)**	
nodefactor MENAT			−0.29(0.14)*	
nodefactor Pakistan			−0.37(0.16)*	
nodefactor Somalia			0.15(0.14)	
nodefactor Sub Saharan Africa			−0.24(0.24)	
nodefactor United States			−0.24(0.13)	
nodefactor West Bank and Gaza			−0.26(0.17)	
nodefactor Florida				−0.05(0.13)
nodefactor Michigan				−0.64(0.13)***
nodefactor Minnesota				0.25(0.12)*
nodefactor New Jersey				0.11(0.13)
nodefactor New York				−0.14(0.11)
nodefactor North Carolina				0.12(0.14)
nodefactor Ohio				−0.11(0.18)
nodefactor Texas				−0.36(0.14)*
nodefactor Virginia				0.29(0.11)*
nodefactor gender		−0.01(0.24)	0.03(0.25)	−0.06(0.26)
nodefactor 2006 to 2010		−0.09(0.03)*	−0.16(0.04)***	0.02(0.05)
nodefactor 2011 to 2015		−0.25(0.09)**	−0.39(0.10)***	−0.21(0.11)
nodefactor 2016 to 2020		−0.56(0.14)***	−0.61(0.15)***	−0.38(0.16)*
nodefactor before 2001		0.47(0.12)***	0.34(0.12)**	0.49(0.14)***
AIC	11241.90	6413.38	6242.96	5936.07
BIC	11251.61	6607.57	6543.96	6275.92
Log Likelihood	−5619.95	−3186.69	−3090.48	−2933.03

*** *p* < 0.001;

** *p* < 0.01;

* *p* < 0.05

The parameter estimates are displayed as log odds and indicate the strength and direction of network patterns. A positive (negative) estimate indicates more (less) of the configuration in the network than expected (given the other effects in the model). Categories “unknown” and “other” are controlled for but not reported.

**Table 3:** ERGM comparisons of the religious converts and family and friends models (M5 and M6).

	M5 Estimate (SE)	M6 Estimate (SE)
**Purely structural effects**		
*Transitivity*		
gwesp (fixed 0.25)	3.82(0.13)***	3.77(0.13)***
*Controls*		
edges	−10.21(0.33)***	−10.65(0.34)***
gwdeg (fixed 0.25)	2.61(0.20)***	2.70(0.20)***
**Actor-relation effects**		
*Homophily (key terms)*		
nodematch terrorist group affiliation	1.55(0.06)***	1.56(0.06)***
nodematch religious convert	0.31(0.08)***	
nodematch family members		0.18(0.07)**
nodematch friends		0.55(0.06)***
*Controls*		
nodematch gender	0.27(0.27)	0.23(0.27)
nodematch year of exposure	0.85(0.05)***	0.83(0.05)***
nodefactor al-Shabaab	0.18(0.04)***	0.22(0.04)***
nodefactor Hamas	0.28(0.08)***	0.34(0.08)***
nodefactor Hezbollah	0.28(0.04)***	0.32(0.05)***
nodefactor ISIS	0.16(0.09)	0.06(0.10)
nodefactor no formal org.	0.16(0.15)	0.18(0.16)
nodefactor Taliban	0.42(0.06)***	0.43(0.06)***
nodefactor religious convert	0.08(0.05)	
nodefactor family members		0.15(0.04)***
nodefactor friends		0.37(0.04)***
nodefactor gender	−0.01(0.25)	−0.03(0.25)
nodefactor 2006 to 2010	−0.09(0.04)*	−0.08(0.04)
nodefactor 2011 to 2015	−0.24(0.09)**	−0.23(0.10)*
nodefactor 2016 to 2020	−0.56(0.14)***	−0.47(0.15)**
nodefactor before 2001	0.48(0.12)***	0.52(0.12)***
AIC	6395.96	6281.03
BIC	6609.58	6514.06
Log Likelihood	−3175.98	−3116.51

*** *p* < 0.001;

** *p* < 0.01;

* *p* < 0.05

The parameter estimates are displayed as log odds and indicate the strength and direction of network patterns. A positive (negative) estimate indicates more (less) of the configuration in the network than expected (given the other effects in the model). Categories “unknown” and “other” are controlled for but not reported.

The algorithm did not converge when fitting a model that included *terrorist group affiliation*, *country of origin*, and *location of exposure*, together with the purely structural variables in the same model. It is well known that ERGMs are quite difficult to fit, especially when purely structural effects, such as *gwesp* and *gwdegree*, are included [87]. We produced goodness-of-fit statistics for all the models included in the paper. We used the gof() function [85] for this, relying on the test proposed by Hunter et al. [84]. We compared the following network statistics across the simulated networks to those of the observed networks: calculated degree statistics and edgewise shared partner. Since these structural aspects were important to our analyses, we used them to diagnose the goodness-of-fit of the ERGMs and to assess how well the models managed to capture features of data. Finally, we assessed the multicollinearity by checking the Variance Inflation Factors (VIFs) of all the models reported in the results section.

#### Robustness checks

We performed checks to confirm the robustness of our findings (see S2 and S3 Tables in S2 File). First, the probability of forming ties between two Islamist extremists might be conditional on the fact that they shared the intention to carry out a violent plot within the United States. In supplementary analyses (S2 Table in S2 File, model R1), we estimated the baseline model M2 controlling for the fact that the extremist planned or carried out an illegal ideologically motivated violent action to take place within the United States. Second, we could not achieve model convergence by estimating an ERGM that included the purely structural effects *gwesp* and *gwdegree* as well as all the actor-relation effects *country of origin*, *terrorist group affiliation*, and *location of exposure* in the same model. However, in supplementary analyses (S3 Table in S2 File, model R2), we estimated a model that included the actor-relation effects, but without the purely structural effects. The purpose of this analysis was to establish whether the effects of *country of origin*, *terrorist group affiliation*, and *location of exposure* remained robust when these variables were all included in the same model. Third, as Fig 1 demonstrates, certain terrorist groups were more active at some time periods than others. For example, by 2014, ISIS had established itself as the leader of the global jihadist movement, while al-Qaeda had been decimated by years of US military interventions in Afghanistan and the tribal areas of Pakistan, and a robust counterterrorism strategy in Yemen. Individuals who radicalized after ISIS established its caliphate in Syria and Iraq naturally gravitated to the group over other jihadist organizations because it was largely perceived to be the most successful Salafi jihadist group operating around the world. From 2016 onward, almost all Islamist extremists radicalized in the US were affiliated with ISIS. In supplementary analyses (S3 Table in S2 File, model R3), we tested whether the findings still hold when collapsing fewer years of co-offending data. To this purpose, we estimated the results presented in R2 on a subsample of 382 offenders (with 756 connections). This sample excluded Islamist offenders radicalized after the year 2015. Initially, we planned to run this robustness check on our baseline model M2 (Table 2). However, when excluding the cases after 2015 the algorithm was no longer able to achieve model convergence for M2. Fourth, in models R4 and R5 (S3 Table in S2 File), we assessed whether the effects of the variables *religious converts*, *family members*, and *friends*, were altered upon their inclusion in the same model alongside *country of origin*, *terrorist group affiliation*, and *location of exposure*. Once again, these models did not include the purely structural effects.

## Results

### Network structure and communities

**Fig 2:**
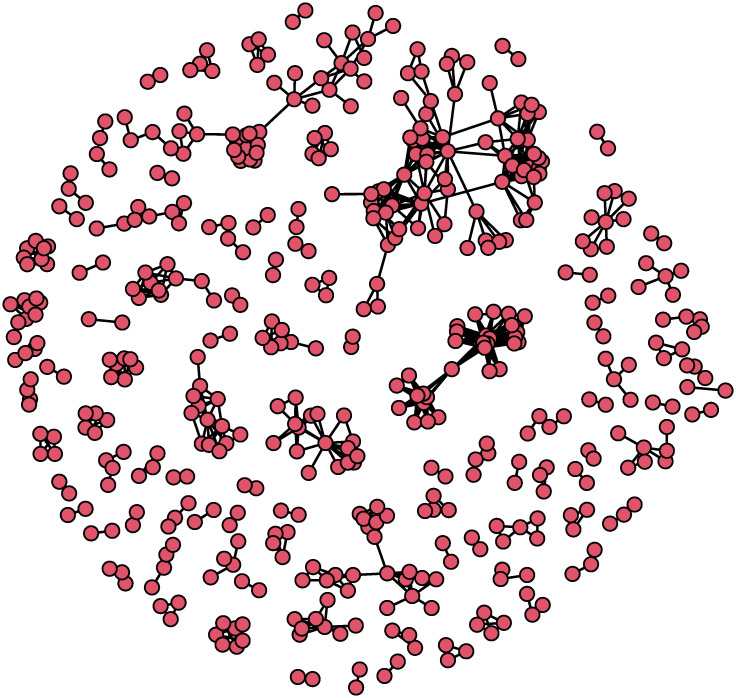
US Islamist co-offending network. Note: isolated nodes are excluded. The network includes only individuals who had at least one co-offending relationship.

Fig 2 shows the US Islamist co-offending network. The network was characterized by a low network density (.008) and a high network transitivity (.706).

**Fig 3:**
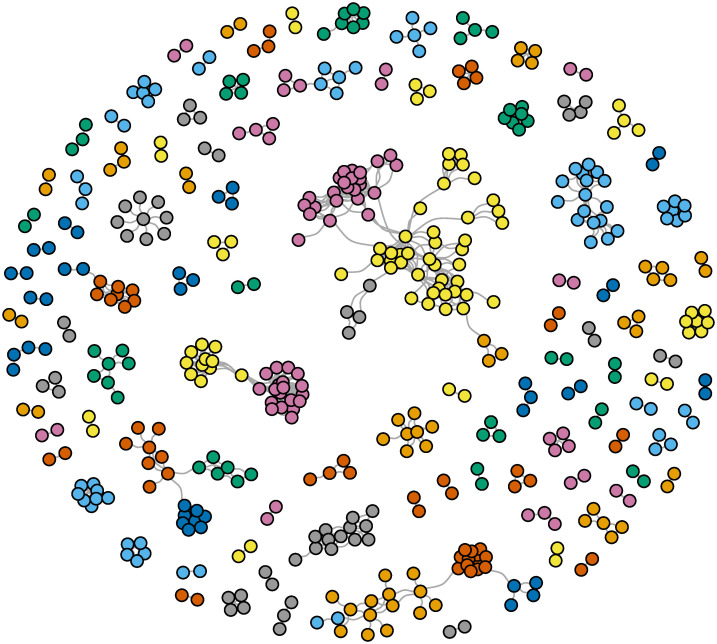
Communities within the US Islamist co-offending network. Note: nodes are color-coded according to the community membership.

Fig 3 shows the result of the community detection in the US Islamist co-offending network. The Walktrap community detection algorithm identified 117 distinct communities (for a detailed report of the individuals included in each community, see S1 File). The modularity score based on the Walktrap achieved very high values (.926). The results from the Louvain community detection algorithm revealed a similar number of communities and achieved a comparably high modularity score. According to these results, the US Islamist co-offending network had very dense connections between members of the same cluster, but sparse connections across clusters.

The largest community (31 members) in the US Islamist co-offending network was made of individuals belonging, with one exception, to the al-Shabaab terrorist group. Around 90 percent of these individuals radicalized in Minnesota between 2007 and 2015. Roughly 81 percent were from Somalia. Three out of the 31 group members were females, and only four were religious converts. Only five were involved in plots to commit terrorist attacks on US soil that did not succeed. None of the individuals were family members, but 23 percent had friends in the network.

The second largest community (24 members) bore some resemblance with the largest community. Again, with one exception, the individuals in this community radicalized in Minnesota and only two were religious converts. Around 92 percent immigrated from Somalia. However, this was an all-male community, and the members of this community were affiliated with, or inspired by ISIS. Only three members of this community were affiliated with al-Shabaab. They also radicalized later, between 2012 and 2016. None of the members were involved in US terrorist plots, but all (and some successfully) attempted to travel outside the US to fight abroad. Most interestingly, this community included many family and friendship relations. Around 63 percent had family members, and 83 percent had friends in the US Islamist co-offending network. Together, the two largest communities included 11 percent of the members of the US Islamist co-offending network (55 nodes) and made up 24 percent of all the co-offending relationships (233 edges) in the network.

The six medium-sized communities included 11 to 19 members who belonged to specific terrorist groups, except for one community that included members of different terrorist groups. Members of two communities were exclusively affiliated with al-Qaeda, the other two communities included only members of Hezbollah, and in one community individuals belonged only to Hamas. In two communities, all members were radicalized in the same year. In the other communities, they radicalized in a time span ranging from four to seven years. Four of the six medium-sized communities included primarily members who radicalized in the same or adjacent states. Two of these communities included prevalently individuals from the same countries of origin, the West Bank and Gaza Strip and Lebanon, respectively. The other four communities included individuals from several countries of origin. The percentage of converts in the communities varied widely, from 0 to 40 percent. In one of these medium-sized communities, 40 percent had friends in the network, but in all others, individuals had no friends in the network. Between 15 to 39 percent of the members of the medium-sized communities had family members in the network.

A closer look at the 117 communities revealed the following patterns. First, around 7 percent of the Islamist extremists in the network were females. They were distributed across 24 communities. In 75 percent of these communities, there was only one female present. No community featured more than three women. Second, Islamist extremists originating from Somalia, the West Bank and Gaza Strip, and Lebanon tended to cluster together in larger communities, where they represented over 80 percent, and in some cases over 90 percent, of the community members. Conversely, Islamist extremists who were born in the United States typically only built homogeneous clusters with other co-citizens in small communities of two to four members. Third, the findings also showed that for some terrorist groups, the members clustered in large numbers in the same communities. For example, one community assembled 53 percent of all individuals affiliated with al-Shabaab. Another included 76 percent of all individuals linked to Hamas. And yet another included 39 percent of Hezbollah members present in the US Islamist co-offending network. Members of the two larger terrorist groups—al-Qaeda and ISIS—were spread around different communities in smaller numbers. Fourth, around 76 percent of the communities were made up entirely of individuals from the same state. Moreover, depending on the state where the individuals were radicalized, they clustered together in the same community or were spread across different communities. For example, 82 percent of individuals from Minnesota were equally distributed in two larger communities. Conversely, Islamist extremists from New York were spread across different communities. Fifth, around one-fourth of the members of the US Islamist extremist network were religious converts. Seventeen communities included exclusively converts. However, except for one larger community consisting of seven members, communities made up of converts typically only included two to three members. Sixth, the results also revealed that individuals who were friends before radicalization tended to cluster in the same communities. The second largest community was made out almost entirely of friends. Friends oftentimes gathered in communities of seven to eight members, but fewer friends were present in communities of 6 or fewer members. Family members also typically tended to cluster in the same communities. Around 34 percent of the dyadic communities were among two individuals who were family members.

### Transitivity, homophily, and probabilities of forming co-offending relationships

The estimation algorithm converged on all six models presented in Tables 2 and 3.

We started by investigating the purely structural effects. The results from Tables 2 and 3 indicated that the parameter estimate for transitivity (*gwesp*) was a robust predictor of forming co-offending relations in the US Islamist co-offending network. In all the estimated models, the estimate was positive and statistically significant. This result suggested a tendency towards triadic closure in the US Islamist co-offender network. We also found a positive and significant effect for the parameter estimate for *gwdegree*. By controlling for these parameters, we were able to make principled inferences about the actor-relation effects in the US Islamist extremist network.

The results from the ERGM analyses highlighted the robust actor-relation effects of the homophily estimates for *terrorist group affiliation* (M2), *country of origin* (M3), and *location of exposure* (M4). The positive and significant effects of these estimates indicated that homophily influenced the formation of co-offending ties. Furthermore, they suggested that Islamist extremists in the United States were substantially more likely to engage in co-offending relations with other Islamist extremists when they belonged to the same terrorist group, shared the same country of origin, or were radicalized in the same US state. In M5 (Table 3) we tested whether in the US Islamist co-offending network Islamist extremists were more likely to co-offend with whom they shared the same status of religious converts. The estimate was positive, indicating that converts and non-converts were more likely to co-offend within their respective groups. Model M6 (Table 3) also indicated tendencies to form co-offending relationships among offenders who shared the same status of having (or not having) family members or friends in the network. Finally, the homophily estimate for the *year of exposure* was consistently and positively associated with a higher tendency to form co-offending ties, pointing to the importance of controlling for this variable in the analyses of the US Islamist co-offending network. Conversely, the results for the homophily estimate for *gender* showed that there was no higher within-group tendency for co-offending within the same gender once we included other relevant homophily estimates (M2 to M6) in the analyses.

**Fig 4:**
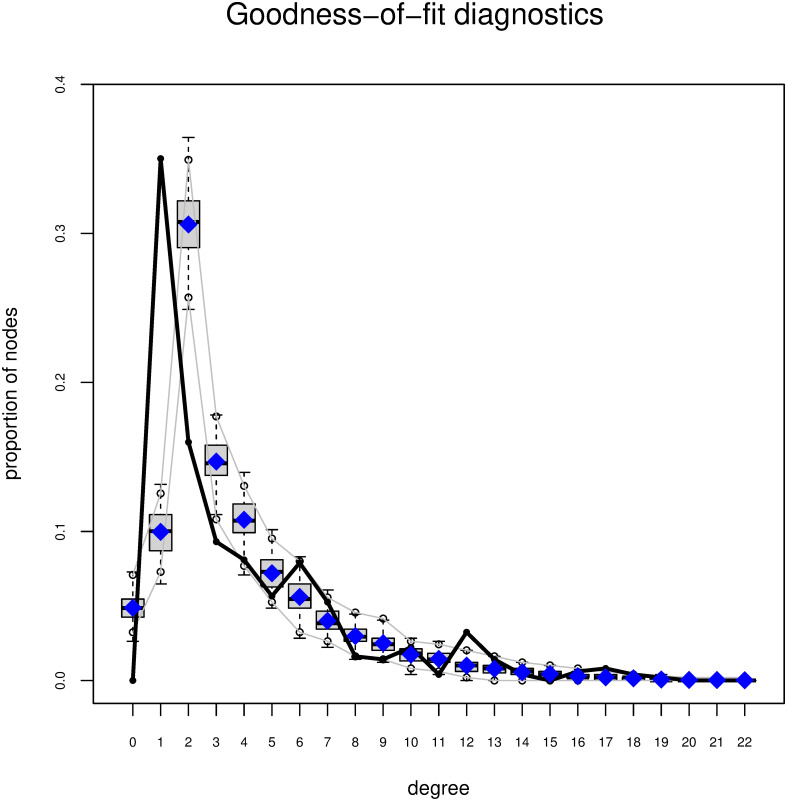
GOF degree statistics. Note: the boxplots represent the values of the simulated networks. The black line shows the observed statistics in the actual network.

**Fig 5:**
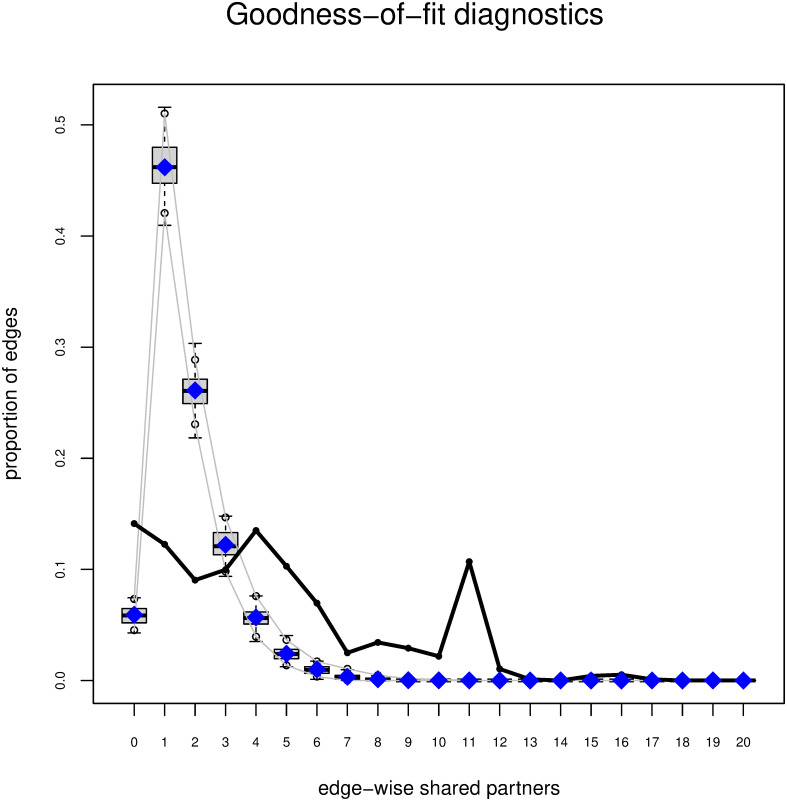
GOF edgewise shared partners statistics. Note: the boxplots represent the values of the simulated networks. The black line shows the observed statistics in the actual network.

The results from the VIF analyses indicated that the values were within acceptable boundaries (the VIF values were generally low and all below the threshold of 20) for all the models reported in Tables 2 and 3, indicating that multicollinearity was not a major concern. Figs 4 and 5 show the results from the goodness-of-fit (GOF) statistics for model M4. We simulated from the model to generate a distribution of graphs. Compared to M1 where we controlled only for the *edges*, the GOF degree and edgewise-shared partner statistics of M4 improved substantially once we included the purely structural and actor-relation effects. We achieved comparable results for the models M3, M5, and M6.

The results from Fig 4 indicate that M4 was well able to capture the degree statistics. Conversely, for the edgewise shared partner statistics (Fig 5), in some instances, the observed values considerably deviated from the mean of the statistics in the simulated network. First, our network excluded Islamist extremist offenders who had no co-offending relationship with other offenders in the network. Therefore, our network had no nodes with a degree of 0. Second, the Islamist extremist co-offender network included a high number of pendants, nodes that were connected to other nodes with a single connection. Third, the network featured some larger clusters that were highly interconnected. These were characteristics of our network that likely differentiated it from the simulated networks and influenced the observed network statistics.

We checked for the robustness of the findings and computed the analyses controlling for involvement in plots carried out in the United States, see S2 Table in S2 File model R1, and did not record any significant changes in the results.

Findings from the community detection suggested that the *location of exposure* had some overlaps with *terrorist group affiliation* and *country of origin*. For example, most of the Islamist extremists from Minnesota came from Somalia and were affiliated with al-Shabaab or ISIS. In the supplementary analyses reported in S3 Table in S2 File model R2, we estimated ERGMs including the three homophily covariates but excluding the purely structural effects *gwesp* and *gwdegree*. The three homophily estimates continued to be positive and statistically significant. We also tested whether the findings continued to hold when collapsing fewer years of co-offending and excluded the years after 2015 in S3 Table in S2 File model R3. The *nodematch* values for *country of origin*, *terrorist group affiliation*, and *location of exposure* remained positive and statistically significant. Finally, the variable *religious convert* was highly associated with the *country of origin* (see S1 Table in S2 File). Most of the religious converts included in the database originated from the United States. Indeed, when including this variable in the same model as *country of origin* (S3 Table in S2 File, model R4), the effects of *religious convert* were fully mediated. Conversely, the effects of *friends*, and *family members* did not change substantially when including all actor-relation effects in S3 Table in S2 File model R5.

## Discussion

Islamist extremists are known for forming covert networks that engage in highly lethal violence [21, 22]. Yet, little is known about why some Islamist extremists form co-offending ties with each other while others do not. Research [16–18] suggests that illicit groups favor network structures that increase security more than those that improve efficiency. These structures are typically characterized by high levels of transitivity [69].

We argue that alongside transitivity, homophily—the tendency of individuals to connect based on their shared characteristics—is the primary mechanism through which Islamist extremists form ties that improve the overall security of their networks. Homophily, which has been found to drive connections in a variety of social networks [90–93] produces network ties that are based on shared norms and values, common experiences, and mutual trust.

We have investigated the structure of the communities in the network of US Islamist co-offenders, as well as the roles that homophily and transitivity play in driving the creation of co-offending ties, using a new database on the co-offending relationships and individual attributes of US Islamist extremists who committed criminal offenses through 2020. In our analyses, we rely on community detection algorithms as well as ERGMs that control for gender and the year the individual was arrested or committed a terrorist attack. Our findings point to a highly security-oriented network, in line with observations from previous research on extremist networks [15, 16]. We find that the structure of the US Islamist co-offending network is characterized by a high level of clustering and low density. The community analyses support these findings and point to a highly modular network. Relative to the size of the network, the algorithms identify many small close-knit communities that are disconnected from each other. These findings support observations from other studies [7, 93] pointing out that violent extremist organizations do not operate hierarchically, but are rather organized in patterns of decentralized, self-governing hubs—a characteristic that renders them particularly resilient to disruption. Research [94] found that such dense cohesive clusters facilitate within-group coordination and increase group compliance.

Next, findings from the ERGMs indicate that there are significant purely structural and actor-relation effects in the US Islamist co-offending network. That is, both transitivity and homophily help explain the presence of co-offending ties. The purely structural effects (i.e. transitivity) by themselves do not cancel out the actor-relation effects (i.e. homophily), highlighting the importance of examining the individual attributes of the offenders in the network. Furthermore, the inclusion of individual attributes does not cancel the purely structural effects found for the network, indicating that individual attributes alone are insufficient for explaining the formation of co-offending ties.

Furthermore, the results emphasize that in the US Islamist co-offending network, offending occurs around similarity: co-offending network ties are dependent on the shared individual characteristics of actors in the network.

First, we have demonstrated that kinship, friendship, common socio-cultural background, spatial proximity, and terrorist group affiliation are key drivers of the formation of co-offending relationships among US Islamist extremist offenders. We find that individuals who are members of broader communities with shared socio-cultural backgrounds, tend to co-offend. Moreover, the co-offending communities in the US Islamist extremist network are clustered around individuals who share the status of having friends or family members in the network. Islamist extremists who originated from Somalia, the West Bank and Gaza Strip, and Lebanon form the most substantial co-offending networks in the United States. Shared socio-cultural experiences not only facilitated co-offending relationships among those in diaspora communities, but they are also common to the offenders who were born in the United States. Indeed, our results indicate that religious converts, who were mostly born in the United States, rarely form co-offending ties with fellow Islamist extremists who are first- or second-generation immigrants. Rather, converts often cluster together to form their own small, close-knit co-offending communities. As outsiders with different socio-cultural backgrounds and experiences, religious converts may not have extensive personal interactions with members of immigrant or diaspora communities to facilitate co-offending, and members of both groups are likely to view each other with greater degrees of suspicion than they would members of their own communities. As a result, converts often seek out each other when looking to form ties with other Jihadists. However, unlike diaspora communities that are typically concentrated in particular cities or regions of the United States, the converts in the data are more geographically dispersed, resulting in small, disconnected communities. Overall, these results support findings from previous research suggesting that familiarity through kinship and common background are important aspects of forming secure relationships in co-offending networks [26, 95].

Second, we find that Islamist extremists tend to favor co-offending with other fellow extremists who are radicalized in the same state. Indeed, more than three-quarters of the Islamist co-offending communities that we have detected are comprised of individuals who reside in the same geographic areas of the United States. Research analyzing other types of co-offending networks [41, 53] has reached similar conclusions. The effects of location of exposure remain robust when controlling for ideological affiliation, and year of exposure. This finding suggests that while online communities can foster relationships among like-minded extremists, co-offender communities tend to form locally [96] and are often comprised of individuals who are linked through family, friendship, or other real-world experiences [95]. Local connections and face-to-face interactions produce a level of trust between potential co-offenders that is difficult to achieve online, where people can easily misrepresent themselves and their intentions. Thus, while online extremist communities are important for disseminating content and promoting extremist beliefs, the mobilization of offenders is more likely to occur in tight-knit, locally based offender groups.

Our analyses identified two large, highly connected communities in the US Islamist co-offending network that are located in the same part of the United States. These communities include members of the Somali diaspora community that settled in Minneapolis, Minnesota, beginning in the early 1990s. The members of these co-offending communities are almost exclusively connected to al-Shabaab and ISIS, respectively. The Minnesota communities are characterized by a high number of friendships and kinship ties. The individuals from Minneapolis radicalized in two waves. From 2007–2011, more than two dozen individuals from the network traveled, or attempted to travel, overseas to fight with al-Shabaab. From 2013–2016, a second wave of fighters from Minneapolis was drawn to the civil war in Syria. Most of these network members attempted to join the Islamic State after it declared its caliphate in 2014; although, many were unsuccessful due to heightened security policies in the United States. In addition to the Minneapolis communities, our results reveal smaller but similarly dense and homogenous communities that formed in support of Hamas and Hezbollah. However, they are more varied regarding the location of radicalization and country of origin.

Third, we find evidence that ideological and terrorist group affiliation is key to understanding the US Islamist co-offending network. Our results suggest that these factors matter to a great extent, with members of most Islamist groups being more likely to engage in a co-offending relationship with a member of the same group. For instance, individuals affiliated with, or inspired by, al-Shabaab, Hamas, and Hezbollah have clustered together to form the largest communities in the US Islamist extremist network.

Finally, purely structural effects also have a unique impact on co-offending ties in the US Islamist co-offending network. Indeed, our results suggest that the community structures of the US Islamist co-offending network display a high degree of transitivity. Transitivity reflects the clustering of relationships within a network, and it plays a crucial role in understanding the structural patterns and dynamics of social connections. In the context of co-offending networks, where nodes represent individuals and edges denote instances of joint criminal activity, transitivity can shed light on the propensity for groups of individuals to engage in criminal collaborations. High levels of transitivity in a co-offending network suggest that individuals who have committed crimes together, or assisted each other in committing separate crimes, may be more likely to form interconnected groups or alliances. This network characteristic is valuable for law enforcement and criminologists in identifying not only individual offenders but also cohesive subgroups within the larger criminal network, aiding in the development of targeted intervention and crime prevention strategies.

The goal of our paper was to detect communities and analyze how co-offending relationships form in the network of US Islamist co-offenders, but we acknowledge that this research represents an initial attempt to understand these relationships and we offer several avenues for future research. First, we do not know whether our results can be generalized outside of the United States. While there is research on foreign fighters and attack plotters from Europe [30] that suggests that local networks based on shared socio-cultural experiences and group affinities have been important in the formation of co-offending ties, more work could be done to apply SNA to the study of terrorist offending outside of the United States. Second, we do not know if our results hold for networks of violent left- and right-wing extremist offenders in the United States or elsewhere. There is currently a critical lack of network-level studies of domestic terrorist movements; although qualitative assessments find similar geographic and group characteristics among co-offenders linked to US-based extremist groups. Thus, future research should explore whether our results hold across other ideological categories. Third, the results do not account for changes in the co-offending relationships between two Islamist extremists over time, for example because of arrest or death of the involved parties. Future research may address the temporal dynamics of the relationships by adopting longitudinal approaches to study terrorist co-offending networks. Finally, our analysis only considers local networks and does not map the ties of US jihadists to foreign contacts. As a result, the effects of social interactions are likely undercounted. The secretive nature of foreign communications makes comparisons between drivers of co-offending relationships in the US and other foreign networks very challenging. Further research may replicate this study in other parts of the world and address the question of whether distant networks can replace the functions of local ones.

## Conclusion

The analysis of the US Islamist co-offending network has highlighted the importance of common familial, friendship, and socio-cultural backgrounds, local connections, and shared group preferences in the formation of co-offending ties among US Islamist extremists. They point to two mechanisms—transitivity and homophily—by which extremist offenders attempt to form secure relationships and achieve their goals. Overall, our findings support the notion that violence is perpetrated locally in small, close-knit groups that are highly connected with each other but sparsely connected with other groups. They also re-establish the importance of group identification based on a shared ideology.

This study suggests the need for a multifaceted approach to the disruption of covert terrorist networks and the prevention of violence in the United States. From a law enforcement interdiction perspective, our results support previous findings [97] that suggest that traditional local policing techniques rather than elaborate online investigations are often the key to disrupting terrorism plots in the United States. Federal and local law enforcement have expanded the number of resources they are dedicating to monitoring online extremist communities in the hopes of identifying and disrupting terrorist networks [98], but our results suggest that extremists in the United States are far more likely to form co-offending relationships with people in their local communities with whom they share common backgrounds or have had personal interactions. Digital environments are important for spreading extremist content, sharing tactical knowledge, and radicalizing people, but mobilization to terrorism is often a local, face-to-face process that is disrupted through community tips, the use of local informants, and traditional policing. It is important to note that our emphasis on local network dynamics is not a call for greater surveillance of members of particular faith or diaspora communities, which has been shown to produce community backlash, reduce trust in law enforcement, and contribute to the radicalization of extremists [99]. Rather, law enforcement should build partnerships with local communities that are based on mutual respect, trust, and community needs in order to foster an exchange of information and improve their awareness of concerning behaviors in their jurisdictions.

In terms of violence prevention, our results suggest that programs designed to inoculate people against extremist recruitment [100] or to help people disengage from extremist groups must be contextualized and reflect local dynamics, shared socio-cultural experiences, and common grievances. Community-based public health models of violence prevention [101], which emphasize improving community education and dialogue, supporting the development of pro-social relationships and strong families, and providing support services to address individual risk factors and vulnerabilities, are promising options for achieving these goals. While the use of prevention models in online spaces is an important part of stopping the spread of extremist beliefs and preventing radicalization, these efforts, which have grown in recent years, must be paired with local programs that are better suited to disrupt the formation of co-offending ties among community members.

## Supporting information

S1 File Communities detected by the Walktrap algorithm. (CSV)

S2 File Correlation matrix and robustness checks of the ERGM specifications. (PDF)
