# Supplementary material for: Extremists of a feather flock together? Community structures, transitivity, and patterns of homophily in the US Islamist co-offending network
Source: PLoS One. 2024 Jun 5;19(6):e0298273. doi: 10.1371/journal.pone.0298273 (PMC11152292; doi:10.1371/journal.pone.0298273)
Supplement: S2 File — (PDF) [file pone.0298273.s002.pdf]

S1 Table. Correlation matrix (Cramer's V).

	1	2	3	4	5	6
Gender	1	0.2062	0.1304	0.176	0.2125	0.1119
Country of origin		1	0.7138	0.3577	0.3643	0.3699
Religious convert			1	0.2342	0.3061	0.1949
Terrorist group affiliation				1	0.4245	0.4460
Location of exposure					1	0.2930
Year of exposure						1

S2 Table. Robustness checks of the ERGM specifications: US plot involvement.

	R1<br>Estimate (SE)
**Purely structural effects**	
Transitivity	
gwesp (fixed 0.25)	$3.82 (0.13)^{***}$
Controls	
edges	$-9.94 (0.33)^{***}$
gwdeg (fixed 0.25)	$2.88 (0.21)^{***}$
**Actor-relation effects**	
Homophily (key terms)	
nodematch terrorist group affiliation	$1.56 (0.06)^{***}$
nodematch us plot involvement	0.08 (0.07)
Controls	
nodematch gender	0.34 (0.28)
nodematch year of exposure	$0.87 (0.06)^{***}$
nodefactor gender	−0.02 (0.26)
nodefactor al-Shabaab	0.09 (0.05)
nodefactor Hamas	$0.35 (0.08)^{***}$
nodefactor Hezbollah	$0.46 (0.04)^{***}$
nodefactor ISIS	0.14 (0.10)
nodefactor no formal organization	0.18 (0.16)
nodefactor Taliban	$0.36 (0.06)^{***}$
nodefactor 2006 to 2010	−0.04 (0.04)
nodefactor 2011 to 2015	$-0.22 (0.10)^{*}$
nodefactor 2016 to 2020	$-0.58 (0.15)^{***}$
nodefactor before 2001	$0.46 (0.12)^{***}$
nodefactor usplot involvement	$-0.19 (0.06)^{***}$
AIC	6322.43
BIC	6545.76
Log Likelihood	−3138.22

$^{***}p < 0.001$; $^{**}p < 0.01$; $^{*}p < 0.05$

The parameter estimates are displayed as log odds and indicate the strength and direction of network patterns. A positive (negative) estimate indicates more (less) of the configuration in the network than expected (given the other effects in the model). Categories "unknown" and "other" are controlled for but not reported.

S3 Table. Robustness checks of the ERGM specifications: homophily estimates.

	R2<br>Estimate (SE)	R3<br>Estimate (SE)	R4<br>Estimate (SE)	R5<br>Estimate (SE)
**Purely structural effects**				
edges	$-7.26\ (0.51)^{***}$	$-6.63\ (0.53)^{***}$	$-7.10\ (0.55)^{***}$	$-8.06\ (0.53)^{***}$
**Actor-relation effects**				
Homophily (key terms)				
nodematch terrorist group affiliation	$2.59\ (0.10)^{***}$	$1.36\ (0.09)^{***}$	$2.59\ (0.10)^{***}$	$2.57\ (0.10)^{***}$
nodematch country of origin	$0.57\ (0.08)^{***}$	$0.20\ (0.10)^{*}$	$0.57\ (0.09)^{***}$	$0.54\ (0.08)^{***}$
nodematch location	$1.89\ (0.08)^{***}$	$0.98\ (0.10)^{***}$	$1.88\ (0.08)^{***}$	$1.81\ (0.08)^{***}$
nodematch religious convert			0.00 (0.10)	
nodematch family members				$0.17\ (0.08)^{*}$
nodematch friends				$0.60\ (0.09)^{***}$
Controls				
nodematch gender	0.19 (0.29)	0.22 (0.28)	0.18 (0.29)	0.13 (0.30)
nodematch year	$1.22\ (0.08)^{***}$	$1.09\ (0.08)^{***}$	$1.22\ (0.08)^{***}$	$1.20\ (0.08)^{***}$
nodefactor Asia	−0.01 (0.19)	0.05 (0.21)	−0.07 (0.20)	0.02 (0.19)
nodefactor Europe	0.14 (0.20)	$0.56\ (0.20)^{**}$	0.10 (0.20)	0.14 (0.20)
nodefactor Lebanon	$-0.86\ (0.22)^{***}$	−0.32 (0.22)	$-0.92\ (0.23)^{***}$	$-0.84\ (0.22)^{***}$
nodefactor MENAT	$-0.36\ (0.17)^{*}$	0.04 (0.18)	$-0.42\ (0.19)^{*}$	$-0.40\ (0.17)^{*}$
nodefactor Pakistan	$-0.49\ (0.19)^{*}$	−0.05 (0.20)	$-0.55\ (0.20)^{**}$	$-0.48\ (0.19)^{*}$
nodefactor Somalia	−0.04 (0.20)	0.15 (0.21)	−0.12 (0.22)	−0.11 (0.20)
nodefactor Sub Saharan Africa	−0.50 (0.28)	−0.20 (0.30)	$-0.56\ (0.28)^{*}$	−0.54 (0.28)
nodefactor United States	−0.20 (0.17)	0.12 (0.18)	−0.22 (0.17)	−0.22 (0.17)
nodefactor West Bank and Gaza Strip	−0.12 (0.21)	0.07 (0.21)	−0.18 (0.22)	−0.19 (0.21)
nodefactor al-Shabaab	$-0.45\ (0.11)^{***}$	$0.29\ (0.11)^{*}$	$-0.44\ (0.11)^{***}$	−0.13 (0.12)
nodefactor Hamas	$0.37\ (0.17)^{*}$	0.27 (0.18)	$0.35\ (0.17)^{*}$	$0.40\ (0.18)^{*}$
nodefactor Hezbollah	$0.94\ (0.13)^{***}$	$0.61\ (0.18)^{***}$	$0.94\ (0.13)^{***}$	$0.94\ (0.13)^{***}$
nodefactor ISIS	0.15 (0.11)	$0.26\ (0.11)^{*}$	0.17 (0.11)	0.11 (0.11)
nodefactor no formal organization	$0.40\ (0.14)^{**}$	0.23 (0.14)	$0.39\ (0.14)^{**}$	$0.41\ (0.14)^{**}$
nodefactor Taliban	$0.65\ (0.12)^{***}$	$0.47\ (0.12)^{***}$	$0.64\ (0.12)^{***}$	$0.60\ (0.12)^{***}$
nodefactor Florida	−0.11 (0.15)	$0.42\ (0.16)^{*}$	−0.10 (0.15)	−0.06 (0.15)
nodefactor Michigan	$-0.37\ (0.17)^{*}$	0.07 (0.22)	$-0.39\ (0.17)^{*}$	$-0.38\ (0.17)^{*}$
nodefactor Minnesota	$0.38\ (0.15)^{*}$	0.32 (0.17)	$0.37\ (0.15)^{*}$	0.17 (0.16)
nodefactor New Jersey	0.16 (0.16)	0.05 (0.19)	0.14 (0.16)	0.06 (0.16)
nodefactor New York	−0.17 (0.12)	0.11 (0.14)	−0.19 (0.12)	−0.15 (0.12)
nodefactor North Carolina	$0.34\ (0.17)^{*}$	$0.58\ (0.18)^{**}$	0.32 (0.17)	$0.40\ (0.17)^{*}$
nodefactor Ohio	−0.02 (0.18)	0.11 (0.22)	−0.03 (0.18)	−0.04 (0.18)
nodefactor Texas	−0.25 (0.16)	0.12 (0.19)	−0.25 (0.16)	−0.25 (0.16)
nodefactor religious convert			−0.07 (0.10)	
nodefactor family members				$0.25\ (0.07)^{***}$
nodefactor friends				$0.43\ (0.07)^{***}$
nodefactor gender	0.15 (0.27)	0.08 (0.26)	0.14 (0.27)	0.19 (0.28)
nodefactor Virginia	$0.58\ (0.14)^{***}$	$0.54\ (0.16)^{***}$	$0.57\ (0.14)^{***}$	$0.56\ (0.14)^{***}$
nodefactor 2006 to 2010	−0.13 (0.08)	$-0.34\ (0.08)^{***}$	−0.13 (0.08)	$-0.15\ (0.08)^{*}$
nodefactor 2011 to 2015	$-0.44\ (0.11)^{***}$	$-0.48\ (0.10)^{***}$	$-0.44\ (0.11)^{***}$	$-0.44\ (0.11)^{***}$
nodefactor 2016 to 2020	$-0.59\ (0.15)^{***}$		$-0.60\ (0.15)^{***}$	$-0.58\ (0.15)^{***}$
nodefactor before 2001	$0.55\ (0.19)^{**}$	0.10 (0.25)	$0.57\ (0.19)^{**}$	$0.67\ (0.19)^{***}$
AIC	7779.03	7411.37	7782.32	7708.30
BIC	8206.26	7797.56	8228.97	8174.37
Log Likelihood	−3845.51	−3663.68	−3845.16	−3806.15

$^{***}p < 0.001$; $^{**}p < 0.01$; $^{*}p < 0.05$

The parameter estimates are displayed as log odds and indicate the strength and direction of network patterns. A positive (negative) estimate indicates more (less) of the configuration in the network than expected (given the other effects in the model). Categories ”unknown” and ”other” are controlled for but not reported.
